# Applying multiple frameworks to establish effective virtual collaborative teams in academia: a review and recommendations

**DOI:** 10.1080/10872981.2020.1742968

**Published:** 2020-03-30

**Authors:** Gerald E Crites, Andrea Berry, Elissa Hall, Denise Kay, Mohammed K Khalil, Larry Hurtubise

**Affiliations:** a Campus Associate Dean for Faculty Affairs and Development, Augusta University/University of Georgia Medical Partnership, UGA Health Sciences Campus, Athens, GA, USA; b Executive Director, Faculty Life, University of Central Florida College of Medicine, Orlando, FL, USA; c Associate Director for Curriculum and Educational Technology, Mayo Clinical College of Medicine and Science, Rochester, MN, USA; d Office of Medical Education, University of Central Florida College of Medicine, Orlando, FL, USA; e Department of Biomedical Sciences, University of South Carolina School of Medicine Greenville, Greenville, SC, USA; f College of Pharmacy, Adjunct Associate Professor of Biomedical Education and Anatomy the College of Medicine, The Ohio State University, Columbus, OH, USA

**Keywords:** Distance Education, Online Collaboration, Virtual Communities, Faculty Development, Online Learning, Research and Scholarship

## Abstract

Recently, academic health professionals have been increasing collaboration with peers at a distance for activities such as research, scholarship, and faculty development. Novel virtual technologies enable academic professional teams to overcome time and distance barriers to facilitate collaboration, but little research is available to guide academicians on how to effectively organize and manage virtual collaborative teams using these technologies. Based upon a literature review and six years of experience as a virtual collaborative team, the authors use Boyer’s Scholarship of Integration paradigm to identify and critique four models for virtual collaboration. The literature search devised from the four identified models found references that had a theoretical foundation for peer virtual collaboration and have been adopted in some professional context. The authors present a review of this literature, describe the benefits for adapting these models to academic health profession contexts, and provide a reflective critique about the challenges for their adaptation in these contexts. They also provide a hypothetical scenario to exemplify the application of these models for health-care professionals along with important considerations and tips when forming new virtual peer collaborative teams or problem-solving teams who are not optimally functioning.

## A scenario

You are a Full Professor at a medical school in the Midwest. While attending a national conference at a prominent medical education society, you run into a colleague from another medical school. Over lunch, she mentions that there is a growing interest nationally to establish a model for incorporating professional identity practices into medical school curricula. During a morning meeting of an interest group the next day, you propose a long-term project around the topic and solicit 7–8 collaborators from different institutions (to keep within the range for an optimally functioning small team). After the announcement, seven more faculty members from different medical schools identify themselves as interested. The group spends the next 30 min exchanging contact information and briefly brainstorming ideas with the group.

You set up the first teleconference after using an online polling app. The first teleconference meeting is fully attended and the group demonstrated much energy and a number of enticing ideas were generated. You task two people to consolidate the ideas into one document table and circulate for the next meeting scheduled 3 months later. Two months later, a table is circulated by email with requests for modification, but no one responds to the request. For the second meeting, only six people make the meeting and the tenor changes, and the group demonstrates an inability to make choices and differing opinions on how to proceed. The group reaches no decisions.

To re-energize the group, you choose to move the meeting to a monthly schedule and host the meeting via web conference call with video. During the third meeting, eight people join the call and the added video call helped improve the discourse; however, most members could not effectively follow some of talking points sent out by email. Also during this call, there are many diverse ideas for moving forward but no consensus is reached. Two of the more senior members seem to offer more proposals but no one offers to manage the follow-up. For the fourth meeting one month later, only four people are on the call and you again elect to defer actions. After one more video call with only three people attending, you cease follow-up calls for meetings.

Eight months later while attending the same conference, you run into the same eight people and they continue to strong express interest in the project. Confused, you ask if you could follow-up with each member via email to find out what went wrong …

## Introduction

Collaborative research activities across academic institutions are becoming more frequently cited and virtual communities are common vehicles for this purpose [1,2]. Faculty members can improve productivity via networks of professionals outside of institutions but challenges remain about how to implement them [3]. For example, research productivity for some faculty members can be suppressed due to lack of time, inadequate research infrastructure, and the inherent difficulties with providing faculty development for distant faculty members [4,5]. To date, there is scant research about how to enhance team productivity using these tools and how to guide optimal team functioning in virtual spaces [1,2].

To provide a comprehensive set of recommendations for implementing virtual collaborative teams for academic health professionals, the authors (we) applied the paradigm of Boyer’s Scholarship of Integration paradigm through a broad literature search from various professional domains and used a consensus-based decision process to select relevant articles based upon our six-year experience as productive virtual team [6]. Through this process, we have identified four peer collaboration models that may be relevant to academic health professionals who participate in virtual collaboration at a distance for the applications of research, educational scholarship, working committees, faculty development, and teaching strategy meetings. We summarize the existing literature for each model and how each may be relevant for virtual peer collaboration for academic health professionals. Finally, to make these concepts and principles practical, we provide, through a real-world scenario with follow-up, how they can be used to analyze virtual collaborative team functions and provide considerations and tips for building effective peer virtual teams or problem sole teams who are not functioning optimally.

## Methods

The full details of the literature review and article selection processes are provided in Appendix A. In summary, we identified models based on our prior experience and an extensive web search using a broad search strategy initially (snowballing) and then a focused strategy with specific search terms around identified models with databases from academic medicine, business, psychology, and education. We extensively searched the full texts and hand-searched bibliographies until no new models were identified (saturation). Since more modern technologies may provide a different virtual experiences than in the past, we only selected articles that included synchronous (e.g., video and audio computer calls) and asynchronous tools (e.g., blogs, message boards and shared databases) that used Web 2.0 online browser-based services and activities that can provide similar experiences without browsers (e.g., collaborative apps and social media). We used an iterative screening process that applied a relevancy scoring system to identify the most applicable references.

The search and screening processes yielded four relevant models: Communities of inquiry (COI), communities of practice (COP), professional learning communities (PLC) and online collaborative learning (OCL, also known as Collaborativism). Each following four sections provides a brief description of the model, summary of its research in professional applications (including references with health professional/medical educator contexts if available), a critique of its weaknesses, potential applications to academic health-care activities, followed by a ‘return to the scenario’ discussion that analyzes the scenario through the lens of each model for the presented scenario.

## Community of inquiry

Two decades ago the Community of Inquiry (CoI) framework was developed in the context of computer-mediated conferencing to study asynchronous, text-based discussion [7]. The framework consists of three interdependent elements, or presences, which include (a) teaching presence, (b) social presence, and (c) cognitive presence [8,9]. Teaching presence is established through online instructional design, direct virtual instruction, and facilitated discussion [8,9]. Social presence supports community member discourse via the actively managed digital learning environment by promoting affective expression, open communication, and group cohesion [8,9]. Cognitive presence is reflected by learning issue recognition, facilitating its exploration, and allowing application of integratively created solutions [8,9]. The interdependence of these three presences informs learning and the effectiveness of education experiences [10,11].

Studies of online CoIs found they offer avenues for social critical inquiry, higher order cognitive processing, opportunities to use technology for support and mentorship, and an adaptive context to everyday applications. [10–12] Features of successful online Communities of Inquiry include:

- Individual engagement necessary to group function
- Group learning augmented by social presence and collaboration through building mutual trust and confidence over time
- Teacher/Moderator support, guidance, and feedback [12–14]
- Deliberate design and structure for high order learning [9]
- Practitioner inquiry in a context which is immediately applicable and relevant [12]
- Focus on construction of knowledge instead of learning outcomes [15]

CoI studies have identified a few unique limitations for online collaboration. First, there is a need for effective teacher/moderator stewardship at multiple levels that including the arranging online activities and blending of feedback while engaging virtual technologies [12,14,15]. Second, effective learner/participant stewardship is demonstrated by active engagement, open communication, and the development of trust; lack of these group characteristics can impair establishing cognitive presence in the community functioning at a distance [7,9,12]. Third, the hierarchical, role-based teacher to learner relationship may create difficulties with adopting the framework to peers collaborating online; however, a moderator role is usually necessary to ensure team functioning, even if the moderator role is subdued through ‘an illusion of non-mediation.’ [11,16] Fourth, online learners/participants may develop low confidence or autonomy (and thus poor social presence) when the technology engagement is poor [12,16].

As health professions educators collaborate online, the CoI provides a framework for facilitating social knowledge construction around academic and scholarly goals. [9,16,17] Given the diversity of virtual technologies, technical and design issues can be adapted to the variable needs and capabilities of participants from academic health-care contexts[18]. Virtual collaboration tools can also allow archiving activities such as attendance and points of contributions to assure that all team members have a voice, which fits well with the paradigm of the democratic nature of peer networks in academia.

### Returning to scenario

Utilizing the Community of Inquiry (COI) framework to interpret your email query, you diagnose some of the reasons for the group’s failure. Although you employed a deliberate approach to advance discussions early on, the group failed to reach decisions due to a failure to pinpoint areas of discourse from poor social engagement early in the process (socialization was not promoted enough prior to stating deeper work). Later, the implementation of the video calls improved connectivity by revealing body language, improving focus during the discussion, and most felt they were able to engage in discussion seamlessly having the visual cues. It also became clear to you the group had not been given enough time and priority to fully developed the level of trust to establish accountability, which would have been enhanced with socialization followed by assuring that other voices were heard equally as the more senior members. Finally, one member stated that he appreciated the occasional individualized emails for certain communications as it established that you, as moderator, valued his participation and assured presence for all members could be enhanced during later meetings.

## Community of practice

Community of practice (COP) is defined as ‘groups of people who share a concern or a passion for something they do and learn how to do it better as they interact regularly’ [19,20]. In this definition, a community of practice is characterized by a shared domain of interest, a community of members who interact and learn together, and members who identify as practitioners of a shared practice [19]. Virtual or online COPs provide a virtual space to support the traditional notions of a COP and allow members to connect across spatial and temporal boundaries [21].

Six themes emerge in virtual COPs:

- Leadership – establishing leadership roles can motivate members around common goals
- Sponsorship – protected time and adequate resources are required for individual success
- Shared Objectives – provide direction on team member responsibilities and encourages active contributions
- Boundary spanning – sets parameters and objective benchmarks for the community
- Risk-free environment – encourages open expression and testing new ideas and feel a sense of safety
- Measurements – to assess the progress of the COP activities [22].

While the framework has been utilized in multiple contexts, inter-institutional COPs may have problems with group trust and cohesiveness, which may impact the sustainability of health professionals who collaborate with virtual tools [23]. Additionally, keeping members engaged and oriented to the goals of the group is difficult when not provided with face-to-meetings [24]. Virtual COPs bring new challenges to the framework as educators may struggle to find ‘togetherness’ across space and time and build a culture of collaboration and learning [25]. Finally, while COPs include peers, robust and continuous moderation and coordination must occur in order to keep the group active [26].

Virtual COPs have some potential applications for health-care contexts. In a study of nurse educators, the authors found that nurses who roles spanned both clinical and educational found role tensions eased through the flexible communication tools that the online fora allow, providing learning opportunities about other world perspectives and facilitated opportunities for successful collaborative research [24]. In another study of health professionals, the authors found career satisfaction was enhanced when careful virtual tool selection built the sense of community and belonging [23]. Virtual COPs may also provide scaffolding for teams attempting to solve real world and reduce distance barriers to allow engagement between colleagues with unique shared interests that cannot be satisfied within their local contexts.

### Returning to scenario

The email responses from your colleagues reveal several additional insights via the COP framework. One team member felt like they were never heard on the phone call, thus reducing his input for objective selection and contributed to his waning interest. Another colleague felt as though there was no clear direction and too much meandering conversation, and this lack of a clear focus on project goals and benchmarks, along with their poor document sharing processes, led to poor group reflection and editing, and thus, minimal contributions. Another member, who was going up for promotion, was hoping but failed to take the lead with at least one project; it seems that the power dynamics by more senior members were challenging for her to overcome in the online medium and she withdrew her participation at the recommendation of her supervisor. Finally, most members felt that negotiations around group goals were limited by insufficient time with this step and failure to use other virtual tools (e.g., document archiving) to allow those not present to contribute; this led to members feeling less valued and becoming less engaged.

## Online collaborative learning theory

Online Collaborative Learning Theory (OCL) was originally described as a ‘model of learning in which students are encouraged and supported to work together to create knowledge.’ [27] In OCL, learning is defined as the collaborative ‘knowledge building’ process wherein learners develop conceptual knowledge in virtual environments [28]. As such, enhancing learner discourse is critical to the learning process [27]. OCL, more recently referred to as ‘collaborativism’, is also distinguished by the integral inclusion of virtual educational applications and the online media in the learning process. With virtual technologies, learning is not time or place bound but can be mediated by asynchronous, text-based discourse, and online group discussion. Working together online, learners advance and apply new understanding and use discipline-specific terms and processes to solve problems, construct plans or develop explanations [27].

In OCL, discourse represents a critical component for the learning and/or knowledge construction process [27]. Different from discourse in face to face settings, the attributes of optimal OCL discourse are (1) text-based, (2) internet-mediated, (3) one-to-one and one-to-many interactions, and (4) independent of time and place [27]. As such, the level and substance of discourse is impacted by the quality of the available technologies and how those are engaged [27].

Collaborativism includes three intellectual processes [27]:

- Idea Generating – learners share their divergent views about a topic or problem.
- Idea Organizing – learners clarify, consider and challenge divergent perspectives; they identify and organize similarities and differences with stronger versus weaker positions.
- Intellectual Convergence, learners create a collaborative product, generate a solution, develop shared understanding, and manage the conflict of disagreement.

The weaknesses of the OCL model is a lack of focus on resource, cultural and relational aspects that help maintain continuity. Also, the model was designed for the application of classroom contexts, and the power dynamics of teacher/student may be difficult to adapt in peer academic networks.

The OCL model may be relevant to academic health-care contexts since it is focused on the process of group learning using optimized technologies for the task. For example, lower level technologies (e.g., asynchronous posts) can be effective for concept sharing but more advanced/integrative tools (e.g., video calls and online document building utilities) are needed for deeper learning. Virtual tools are also effective tools to allow archives as a snapshot of group knowledge that can be evolved over time.

### Returning to scenario

Your query of group members suggested that the OCL framework may help build a better understanding about what went wrong. The group failed to identify how the online and technical barriers had compromised the idea generation phase. Balancing the need for ongoing, real time discourse with the need for simpler immediate discourse could have been better complemented with asynchronous methods (such as enduring online archived documents, group emails, and blogs). Through these methods, group members can view each other’s responses, provide real time, immediate but thoughtful responses, and allow the group to move more towards organizing ideas. You decide that, if the group is successful at reaching this stage of the process, they could use these easier to access but lower engagement tools along with more robust tools such as synchronous discourse (e.g., video calls) to proceed to the intellectual convergence stage (i.e., knowledge generation and production of a scholarly product).

## Professional learning communities

Derived from systems learning theories such as organizational learning, Professional Learning Communities (PLCs) were formed within the structures of schools or school systems that were failing to address the learning needs for a diverse set of learners [29]. PLCs take advantage of the working knowledge of teachers by having them collaborate in teams to explore, study, and advise changes to teaching and organizational practices [30]. PLCs have the features of:

- A focus on learning needs of students [29,30]
- A culture of collaboration between teachers and between teams of teachers and school administration [29,30]
- Supports for teacher development [29]
- Teams of teachers coalesce around with common challenges [29]
- Group inquiry processes to find solutions to challenges and document [29]
- Having support (time, space, etc.) for the teams to do inquiry [29]
- A focus on outcomes after best practice changes [30]

Online PLCs can offer: More flexibility with people experiencing time and travel constraints, lower costs for professional development activities, more opportunities to diversify individualized professional development goals, ease of sharing and systemizing data, more access to mentors, and better opportunities to archive and study collaboration processes[31]. Successful online PLCs are characterized by diverse member experiences, strong moderator presence, strong group accountability, and opportunities to ‘socialize’ and allow the development of meaningful interpersonal bonds [31,32]. Online PLCs offer more meaningful reflections, and, for other developmental outcomes, online PLCs can be developmentally equivalent to face-to-face (F2 F) PLCs [31,32]. It appears that these developmental outcomes occur whether utilizing mostly synchronous collaboration, such as video conferencing, or asynchronous methods, such as wikis and discussion boards [33–35].

A critique of PLCs is that it has narrowly been applied to k-12 teaching professionals and have not been applied I multiple professional contexts. Also, in many contexts, organizational goals take precedence over individual professional goals so how groups build their own goals through negotiation is not as well emphasized.

Despite this, academic health professionals share common challenges with K-12 teachers, including needs for professional development through peer collaboration, expectations to ‘show results,’ and time/distance barriers [17,29,36,37,38]. Both groups also have to build team unity to assure ongoing trust and productive relationships [30,39]. Virtual technologies allow archiving living documents that represent a reflection of codified group goals that can evolve into a specific product that applicable and relevant for all members. Also, the lack of online presence is often more conspicuous for virtual teams, and this can help assure that all members are committed and contributing and teams have a sense of accountability.

### Returning to scenario

Your email query from the project group shows some interesting results when applying PLC processes. Several members noted the project was a priority for their home institutions, but the group should have established preliminary-targeted ‘products’ that would meet their institutional and individual goals so they could have continued local support. Finally, individual development needs were not explicitly described and archived, so to assure that the project goals were relevant to each group member’s academic context at every stage of the project. Those who stayed with the project team for a few meetings noted the lack of clear capture of discourse and communications with an online repository caused mild frustration and may have contributed poor motivation among different members.

## Implications and recommendations

In order to make the models in this review more practical, the authors provide a consolidated table to connect the various elements described in the article (Table 1). The table is organized around Tuckman’s Four Stages of Group Development: Forming, storming, norming, and performing [40]. During the forming stage, group members begin to develop generalized goals but, since they can be unfamiliar with each other (at least in the context of the group’s work), they can demonstrate attitudes such as skepticism and aloofness [40]. During the storming stage, groups solidify goals and interactions are characterized as conflicts that are resolved through negotiations [40]. During norming, groups develop shared rules and accountability (for both individual and group) and mutual trust leads the team to become functional and work begins [40]. Finally, during performing, group functioning becomes concrete as the deeper work begins and leads to tangible results [40].

**Table 1.** A summary of key considerations for the four virtual collaboration models with practical tips in the context of group stage development

	COI	COP	OCL	PLC
**Forming– The Stage of Group Goal Setting**^[40]^				
Considerations	- Is group brainstorming happening? - Do group members establish social connection via technology? - Have the group ground rules for netiquette been established?	- Do each member’s professional goals align with the group’s? - Is the domain of practice specified and shared by members?	- Is the technology easy to access and use? -Will the selected technology support the three OCL intellectual processes? - Is idea generating happening?	- Do the group goals propose feasible/tangible outcomes/products? - Does each group member have institutional support?
Tip	- Archive brainstorming in an online workspace (e.g. shared drive) - Use online technologies to build personal presence (e.g., emojis, video body language) - Establish guidelines for virtual interactions	- Early on have each member state their expectations for the group (professional practice needs and development/scholarly goals) – virtually archive (e.g. shared document space)	- Use an easily accessible virtual shared space (e.g., social media community) that supports text-based and real time discussions - Clarify when to use asynchronous virtual collaboration methods are sufficient (e.g., emails, app texting) and when synchronous methods (e.g., video) are needed	- During brainstorming, begin to link potential group goals to important overarching results- archive them (e.g., shared drive or document repository) - Clarify how each member can commit to group activities given local limitations (including local support)
**Storming– The Stage of Group Negotiations for Expectations**^[40]^				
Considerations	- Has the virtual moderator role been defined and assigned (whether fixed or rotating)? - Is the virtual moderator capable of establishing a virtual moderator presence? - Are individual roles emerging or defined?	- Do group members engage each other regularly to facilitate negotiations? - Are parameters for what group activities are important to meet negotiated goals (and what activities are not important)?	- Have early challenges with using online discourse technologies been explicitly addressed? - Has the group negotiated expectations for individual contributions and are they understood?	- Will the negotiated outcomes/products of the inquiry meet the individual and institutional needs?
Tip	- Moderators should confirm his/her comfort level with engaging targeted technologies - Moderator leads the group to negotiate individual roles - Have individuals set his/her proposed contributions (timelines; participation, etc.)- virtually archive (e.g. shared drive)	- Moderator given authority for calling (or scheduling) synchronous meetings (real-time audio and/or video) - Archive online (e.g., shared drive) parameter negotiations for future reference including group milestones	- Acknowledge individual struggles with virtual technology for discussions and provide resources/help (e.g., local IT support) - Archive online (e.g., shared drive) the milestones for all group members to access the shared archives and prepare their contributions – organize fold structures for easy access	- Check-in with each member to assure groups intended outcomes/products align with developmental/scholarly/teaching needs – continually refer to online archives (e.g., shared drive or community blog)
**Norming– The Stage of Group Trust and Accountability**^[40]^				
Considerations	- Has the group engaged technologies that enhance social presence?	-Are the moderator(s) and group members maintaining a safe virtual environment for individual expression? - Is the group becoming interpersonal (to enhance trust)?	- Do group members know how to best contribute to the group discourse with technology and are accountability mechanisms established? - Is there evidence of substantive contributions and idea organizing in group member’s virtual discourse?	- Is there a virtual place where each member can go to be reorient his/her tasks to the overall goals? - Does the group remain committed to the long term goals and the targeted outcomes?
Tip	- Assure technologies always build social presence to task level (e.g., engaging video for high stakes group tasks vs audio/emails only for lower stakes tasks) - Celebrates small, short term victories and show appreciation for members for their contributions (via text or video presence)	- Assure that each member has his/her virtual ‘moment’ during discourse; Positive reinforcement through virtual expressions (e.g., using text, emoji, verbal) - For complex conflicts, group uses video calls when feasible to reduce misunderstanding - Provide virtual ‘social time’ moments – (text vs real-time audio/video discourse)	- Use a task list with assignments (e.g. project management app) -Use frequent, brief check-ins to assure that virtual messages are received and understood. - Frequently check-in to assure members are using the selected discourse platform- assure platform meets needs of the task (e.g., text based apps for basic communication vs real-time technologies for deeper discourse)	- Build/revise online archives where continuously negotiated goals and targets are recorded and clarity is reinforced- date stamp and/or order them by time (building a virtual history of activities) - Moderator frequently restates what the group is trying to accomplish (end game) during specific intervals – refer to archives
**Performing– The Stage of Group Productivity**^[40]^				
Considerations	- Is the knowledge generated by the group effectively converged into singular messages and expressed to all members for internal validation (cognitive presence)?	- Can the group knowledge generated be externally validated with intended results (e.g., scholarly works) - When group is stuck, does it manage the barriers effectively?	- Does the selected technology promote intellectual convergence? - Are there interaction threads/conversations between members that reflect a discourse??	- Are the outcomes/products of inquiry being valued by each member (and his/her institution) and/or external audiences?
Tip	- Utilize online tools (e.g., document archives) that allow real-time and asynchronous group knowledge syntheses - Use integrative or multiple technologies that allow analyses and critique of knowledge syntheses (e.g. real-time video calls while utilizing real-time shared editing tools)	- Check-in at intervals to assure the archived outcomes are moving towards group goals- renegotiate when necessary - When stuck, a group’s options include: reevaluate technical issues; invite new/junior members to join; reestablish timelines; reconsider overarching goals; dissolve group	- Acknowledge when virtual technologies are or are not helping with discourse- resolve with other options (i.e., more integrative approaches- e.g., social media with multiple communication abilities) - Moderator promotes an honest and critical reflection of archived products - Assure that archives have tools that are effective for organizing themes evolving from discussion threads and real-time conversations	- Maintain virtual tools and repositories that can be shared and utilized by all members to monitor progress (e.g., online research tools and databases) - Check-in occasionally to assure that the outcomes/products of the collaboration are valuable to external audiences (e.g., member home institutions; targeted audiences for scholarship, etc.) - Continuously refer and update shared archives to reflect most recent outcomes/products

The table demonstrates the links for each of Tuckmans’ stages across the four models. For each of Tuckmans’ stages, the table highlights important questions (considerations) to address when using one of the models to form a virtual team or to problem-solve when a team is not functioning optimally. We also provide tips to help address each consideration. For some models, there are overlaps in application concepts (e.g., leadership), so the model that best exemplifies this concept is the one represented.

## Discussion

The principles and practices described in this manuscript are a synthesis of literature within and outside of the health-care literature using the paradigm of Boyer’s Scholarship of Integration and our six-year experience as a productive virtual collaborative team [6]. As far as we know, this is the first attempt to integrate different models applied in other professional contexts to academic health-care contexts.

The result of the review process was to identify four models whose principles are most applicable to virtual collaborative teams in academic health education: Community of Inquiry, Community of Practice, Online Collaborative Learning, and Professional Learning Communities. The models had similar themes around sponsorship/support, stewardship and leadership, socialization through a virtual self, choosing technologies to maximize discourse, using multiple virtual tools to enhance deep learning, and establishing a historical archive to codify knowledge gained. Each model also illustrated unique principles that we felt were critical to include, for example the principle of virtual presence from COI, the importance of virtual communication to enhance group negotiations from COP, choosing simpler vs more integrative technologies for scaling knowledge convergence from OCL, and the importance of using the online archiving tools as a method to establish and revise work products from PLC.

The primary weakness of our approach relates to the lack of significant number of contributions from medical education or health professional contexts and, thus, applications to all academic health contexts can be somewhat speculative. Another weakness is the literature has mostly have focused on Web 2.0 technologies that use traditional browser-based services (e.g., video calls, blogs, shared drives, emails, etc.) and little guidance is provided for more novel-based technologies such as collaborative apps and social media (thus the recurrence of the term ‘online’ in the review of the four models as reflective of mainly browser-based technologies). Additionally, the models focused mostly on team design and functioning successes and little research is available to provide best practices to measure team products/outcomes using these approaches. Due to this lack of clarity, we could not perform all activities required for a more intensive review as was intended at the beginning of the inquiry (e.g., scoping review) [41].

Despite these considerations, we do believe this review should provide a framework and recommendations for forming and maintaining successful virtual peer collaborative teams in academic health-care contexts. The imperative for readers to consider using these approaches is that academic teams across institutions and distances are more frequently coming into existence, and their risk of failure is real due to time and distance communication barriers, limited familiarity with interinstitutional peers (new group formation challenges), difficulty with building a socialized team (versus those already established locally), challenges for representing the authentic self during group discourse, variable power relations, and limited local support. The review does provide some guidance about how professionals in healthcare and other contexts have overcome these barriers. Additionally, we believe, based upon our six-year experience, that these models, when matched with effective virtual learning and collaborative tools, may achieve more enduring relationships and more robust knowledge products. We are hopeful that further research will hopefully elucidate more specific approaches for different contexts and how to effectively judge the results and products of these collaborations.

## Figures and Tables

**Figure 1.**
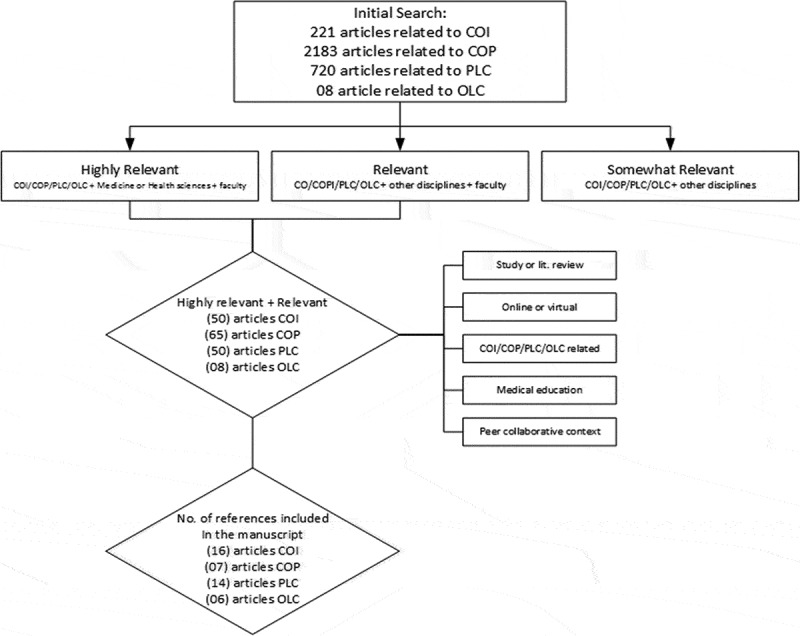
A summary of the search and articles selected for the review (See Appendix A for full search details)

## Appendix A. Review Search and Synthesis Methodologies

Based upon our prior collective experiences, we identified two candidate models. To identify further models, we also conducted a search using broad terms in a web search engine (Google) with combination of terms ‘online,’ ‘web,’ ‘virtual’ with terms ‘teams,’ ‘peers,’ ‘collaboration,’ ‘scholarship’ and ‘research.’ We reviewed citations from database search results (see below) to identify further candidate models and performed additional searches for those identified (snowballing). We repeated iterations until no further candidate models could be identified (information saturation). The electronic databases searched were Ovid Medline, PubMed, Business Source Premiere, ERIC via WorldCat, Education Research Complete, and Business Source Complete. We searched the databases with terms such as ‘online,’ ‘web,’ ‘virtual’ combined with the model name for each database. The author in charge of a particular search could use the unique features of each database (filters and search builders) to further reduce searches with massive results (>2500). Since the focus of the review is academic peers collaborating virtually for professional productivity (research, educational scholarship, working committees, faculty development, and teaching strategy meetings), we intentionally left out terms that would yield applications for student courses/programs and classroom only applications (and excluded citations whose abstracts clearly reflected this).

Through several online research meetings via video calls, emails and a central document repository, we initiated three rounds of screening to improve yield and enhance specificity. For the first round, the author in charge of the search would exclude full abstract citations from the next round if they clearly did not fit the applications described in the paragraph above. For the second round, two authors independently reviewed the full reference and graded their relevance for professional productivity for medical education scholars on a scale of 1–5 (1 – article mentioned some aspect of model, and 2 – for virtual/web applications, and 3 – model was the focus of the manuscript, and 4 – focused on professional learning, planning and development in teams and 5 – for medical education applications specifically). References rated 3 or higher moved to the third screening round. For this round, two independent reviewers rated articles to be included and, when disagreements occurred between the two reviewers, the whole group mediated the disagreement. Also, for this round, references that lacked adequate descriptions for deriving firm inferences or practical applications were excluded. The search and screening commenced January 2017 and ended August 2018.

The search and screening processes are presented in Figure 1 (full strategy details available on request).

The search yielded four relevant models: Communities of inquiry (COI), communities of practice (COP), professional learning communities (PLC) and online collaborative learning (OCL, also known as Collaborativism). The final number of references included in the review were 16 for COI, 14 for PLC, 7 for COP, and 6 for OLC.
